# Amorphous carbon nitride dual-function anti-reflection coating for crystalline silicon solar cells

**DOI:** 10.1038/s41598-022-14078-0

**Published:** 2022-06-14

**Authors:** Ali J. Addie, Raid A. Ismail, Mudhafar A. Mohammed

**Affiliations:** 1 Center of Advanced Materials, Ministry of Science and Technology, Baghdad, Iraq; 2 Applied Science Department, University of Technology-Iraq, Baghdad, Iraq

**Keywords:** Solar cells, Structural properties, Surfaces, interfaces and thin films

## Abstract

Crystalline silicon (c-Si) solar cells have dominated the photovoltaic industry for decades. However, due to high reflectivity and the presence of numerous types of surface contaminants, the solar cell only absorbs a limited amount of the incident solar radiation. To improve the efficiency of the solar cell, anti-reflection and self-cleaning coatings must be applied to the surface. The main objective of this work is to synthesize an amorphous carbon nitride CNx thin film as a novel dual-function anti-reflection coating (ARC) for c-Si solar cells. The CNx film was synthesized by the RF magnetron sputtering technique and characterized by different chemical, structural, and optical analysis techniques. The performance of CNx film was investigated via measuring the reflectance, photoelectric conversion efficiency, and external quantum efficiency. The minimum reflectance was 0.3% at 550 nm wavelength, and the external quantum efficiency achieved was more than 90% within the broad wavelength range. The open circuit voltage and short circuit current density that have been achieved are 578 mV and 33.85 mAcm^−2^, respectively. Finally, a photoelectric conversion efficiency of 13.05% was achieved with the coated c-Si solar cell in comparison with 5.52% for the uncoated c-Si solar cell. This study shows that CNx films have promising application potential as an efficient ARC for c-Si solar cells as compared to traditional ARC materials.

Over the last few decades, crystalline silicon (c-Si) solar cells have enjoyed longstanding dominance and occupied more than 90% of the global photovoltaic (PV) production market^1–4^. This dominance in PV technology has been growing steadily over the last few years because of a combination of abundant materials, long-life stability, and relatively high conversion efficiency, as well as considerable cost reductions, realized via large-scale and well-developed manufacturing processes. Moreover, the fundamental properties of silicon, such as its bandgap (1.12 eV for c-Si), are nearly an ideal match to the solar spectrum, giving c-Si solar cells a dominant advantage over other semiconductor materials for solar conversion. The maximum efficiency of c-Si solar cells is determined mainly by two intrinsic recombination processes occurring within the silicon, namely radiative recombination and Auger recombination. Theoretical calculations show that the fundamental properties of silicon limit the maximum power conversion efficiency of a c-Si solar cell to about 29% under standard operating conditions of air mass 1.5 global solar spectrum (AM1.5G) and 25 °C^5–7^. Typically, calculations assume that both surfaces of the silicon solar cells are roughened, which optimizes optical absorption through light trapping. Until recently, no experimental efficiency records have exceeded the theoretical maximum efficiency limits for c-Si solar cells, as the highest values reported to date do not exceed 90% of the maximum^7^. However, around 35% of the total incident solar radiation losses are due to high optical reflection from the silicon surface of the solar cells, which does not contribute to the photovoltaic energy conversion process, reducing the efficiency of the solar cell. Reflection losses arise due to the impedance mismatch induced by the sudden change in refractive index at the interface from low refractive index air (n = 1) to high refractive index silicon (n = 4). To address this issue, numerous materials and interface designs have been suggested to minimize the reflection losses of c-Si solar cells, including proper silicon surface texturing, subwavelength structures^3,8^, plasmonic nanoparticle surfaces^9^, nanostructured silicon^10^, the surface passivation approach^11,12^, and application of dielectric coating as a single or multi-layer anti-reflective coating (ARC)^13–16^. A dielectric ARC with a thickness of a quarter wavelength (λ/4n) has been widely used to reduce reflections and enhance c-Si solar cell efficiency. The ARC layer creates two interfaces (one with the air and another with the silicon substrate), and hence two reflected waves are generated. Destructive interference occurs when these two waves are out of phase, canceling each other out before they escape the surface. The destructive interference depends mainly on the thickness and reflective index of the coating material. For several decades, titanium dioxide (TiO_2_) was preferred to be used as the ARC for c-Si solar cells because of its refractive index (n = 2) and for chemical stability^17,18^. Today, various materials and thin films are used as ARCs, including Al_2_O_3_^19^, ZnS^20^, ZnO^9,21^, MgF_2_^22^, SiO_2_^13,23^, SiOx^24^, Si_3_N_4_^4,25^, SiNx^26–29^, and SiOxNy^30^. Among these materials, SiNx has become the most preferred ARC for the silicon solar cell because of its superior optical properties, such as its tunable refractive index, which can be ranged between 1.9 and 2.9 by controlling the deposition process^31^. Moreover, SiNx films are characterized by their high-quality surface passivation effect that leads to high bulk lifetimes^26^. Typically, SiNx is deposited by plasma-enhanced chemical vapor deposition (PECDVD). However, the requirement for the process to handle hazardous and flammable gases like ammonia and silane is a major disadvantage because of the high costs involved^31,32^. Numerous research groups have investigated the application of carbon-based materials such as diamond-like carbon (DLC) in silicon solar cells as an ARC^33–37^. The DLC film, however, has encountered difficulties due to its extraordinary internal stress, narrow optical band gap, and high refractive index. Among the several significant studies to improve the anti-reflective properties of the DLC coating, a few suggest that doping DLC with nitrogen may improve the anti-reflective behavior of the coatings, potentially increasing the efficiency of the c-Si solar cell^38,39^.

Amorphous CNx films are of high interest since they have attractive properties due to their chemical composition and structure. CNx films, in particular, may be used as ARC for c-Si solar cells due to its indirect bandgap, excellent mechanical strength, high thermal stability, low reflectivity, and high transmission, as well as their simplicity of fabrication by the reactive magnetron sputtering process^40–43^. This will greatly enhance device performance and the conversion efficiency of c-Si solar cells. However, the application of the CNx film as an ARC for c-Si solar cells has not yet been reported.

In recent years, researchers have been more interested in developing surfaces with anti-reflective, anti-contamination, and scratch-resistant properties because they may suit a variety of requirements in solar cell applications. Therefore, many attempts have been made to fabricate multi-function coating materials^44–48^.

In the present work, the characterization of the CNx thin films and their performance as an ARC were investigated by measuring various chemical, structural, and optical properties. The films are examined using a field emission scanning electron microscope (FESEM) and an atomic force microscope (AFM). The optical band gap, reflectance from the crystalline silicon surface with and without an ARC film, and Raman spectroscopy of the ARC film will all be investigated. After applying an ARC coating to commercial crystalline silicon solar cells, the photoelectric conversion efficiency was determined and compared to that of traditional ARC coatings. Using reactive RF magnetron sputtering, we apply variable deposition parameters to the CNx structure. This technique has an advantage over others in that it allows for precise control of deposition rate and thickness uniformity while using low-cost starting materials and producing no toxic byproducts. We investigated the influence of deposition temperature and time on the properties of amorphous carbon nitride films deposited on glass and silicon substrates.

## Experiments and data analysis

**Figure 1:**
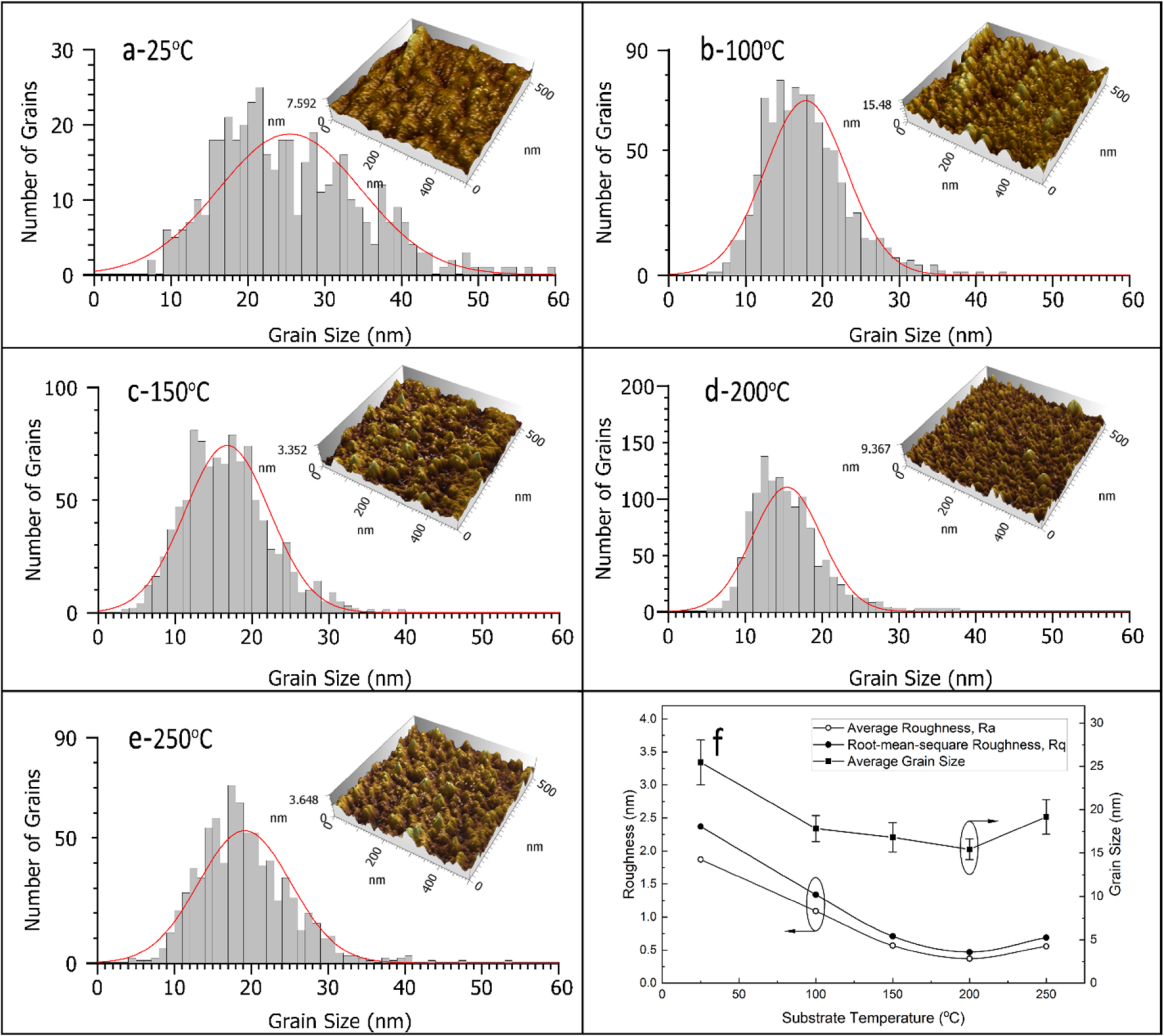
(**a**–**e**) 3D AFM topography images and grain size distributions histogram with fitted Gaussian curves for the coated films produced using a different deposition temperature of: (**a**) 25 °C, (**b**) 100 °C, (**c**) 150 °C, (**d**) 200 °C, and (**e**) 250 °C, and (**f**) variation of grain size, Ra and Rq with substrate temperature.

An AFM was performed to study the surface topography of the CNx coatings. As shown in Fig. 1, the 500 × 500 nm^2^ size 3D AFM surface topography and grain size distribution of the as-deposited carbon nitride thin films on glass at various substrate temperatures are demonstrated. The deposited film has a very smooth surface over the scan area for all samples, with a maximum height of about 15 nm at low substrate temperatures and decreasing to about 3.4 nm at higher substrate temperatures. The uniformity of the grain-like features across the sample surface was reflected in the histogram chart, which displayed a uniform normal distribution. However, the 3D images revealed that aggregated clusters were found over the surface of the films deposited at room temperature. The histogram of the grain size distribution also reveals that the surface features are distributed relatively over a wider range with less uniformity. However, by increasing the substrate temperature up to 200 °C, the films exhibit a more homogeneous and smoother surface topography with a narrower grain size distribution.

The grain size, root-mean-square roughness (Rq), and the average roughness (Ra) estimated from the AFM images were plotted against the substrate temperature in Fig. 1f. As shown in the figure, higher substrate temperatures result in reduced surface roughness and smaller grain sizes. The films deposited at room temperature have the largest grain size, Rq and Ra roughness of 25.5 nm, 2.37 nm, and 1.85 nm, respectively, while the films deposited at 200 °C have the smallest grain size, Rq and Ra of 15.5 nm, 0.46 nm, and 0.37 nm, respectively. As an indicator of the effect of raising the temperature on grain height uniformity and the lack of irregularities on the film surface, the difference between the two roughness parameters, Ra and Rq, was reduced from about 0.5 nm for the lowest substrate temperature to about 0.1 nm for the film deposited at 200 °C.

Generally, the driving force for grain growth is the promoted surface diffusion of the adatoms at a higher substrate temperature^49,50^. However, a different trend was observed in the current study, which might be attributed to a mechanism associated with the reactive sputtering of carbon nitride. According to the structure zone model^51–53^, when the substrate temperature is well below the melting point of the target material, the film will have a very smooth surface and low density. The microstructure will be composed of clusters of small columnar equiaxed grains with a high degree of disorder, or it can be entirely amorphous, which results from the limited mobility of adatoms on the substrate surface. As the temperature of the substrate increases, the adatoms gain more energy and migrate freely over the surface, filling the gap between the columnar structures and reducing surface roughness^54^. As the substrate temperature goes up to 250 °C, the grain size increases significantly. In this case, we assumed that the grain growth contribution at a high substrate temperature would dominate the structure evolution. These results are in good agreement with those previously reported on the effect of substrate temperature on the roughness parameters for the reactive sputtered nitride films^49,55^.

**Figure 2:**
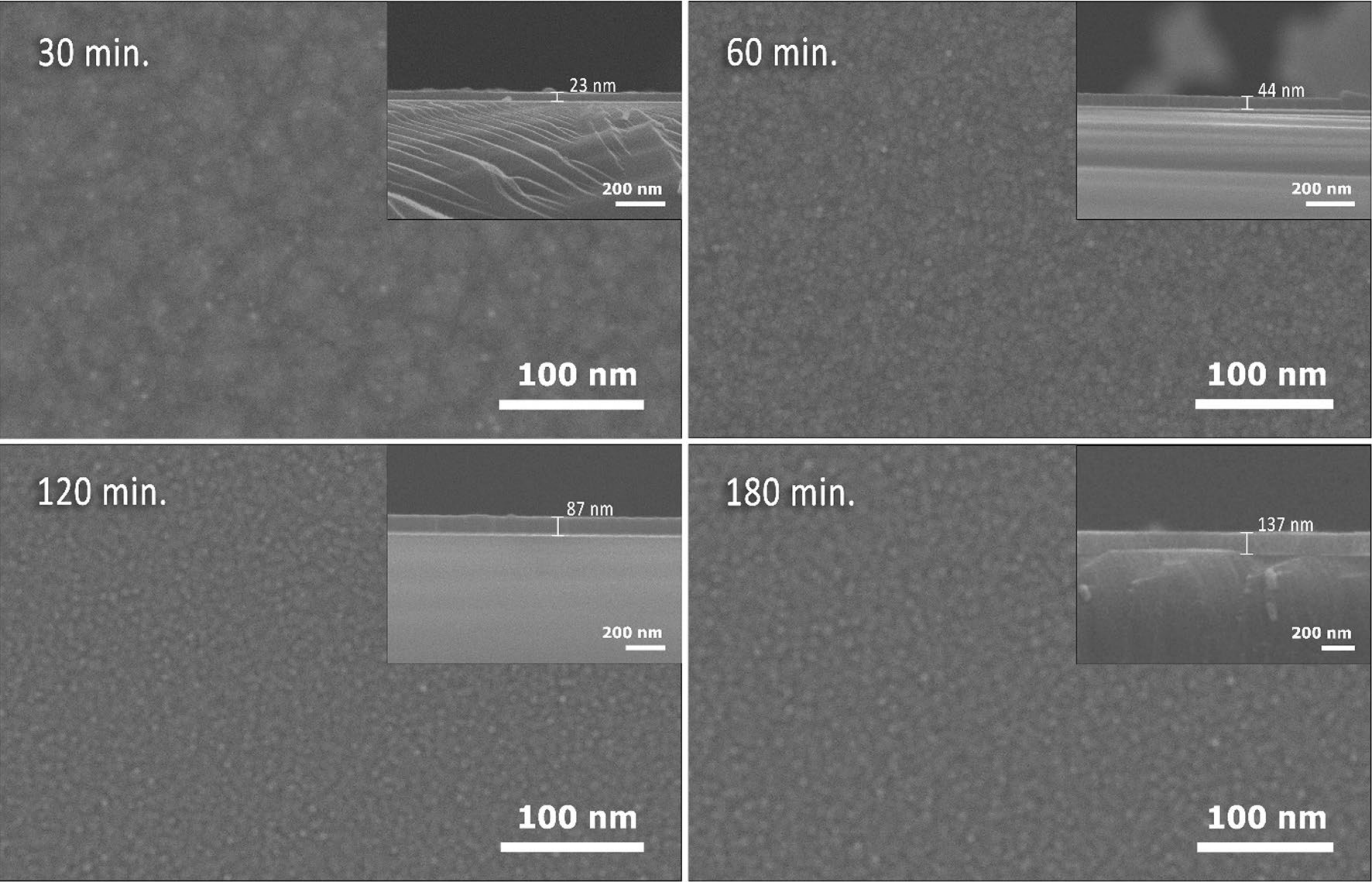
A typical FESEM images showing the morphology and fractured cross-section (as an inset) of carbon nitride films prepared at deposition time of 30, 60, 120, and 180 min.

**Figure 3:**
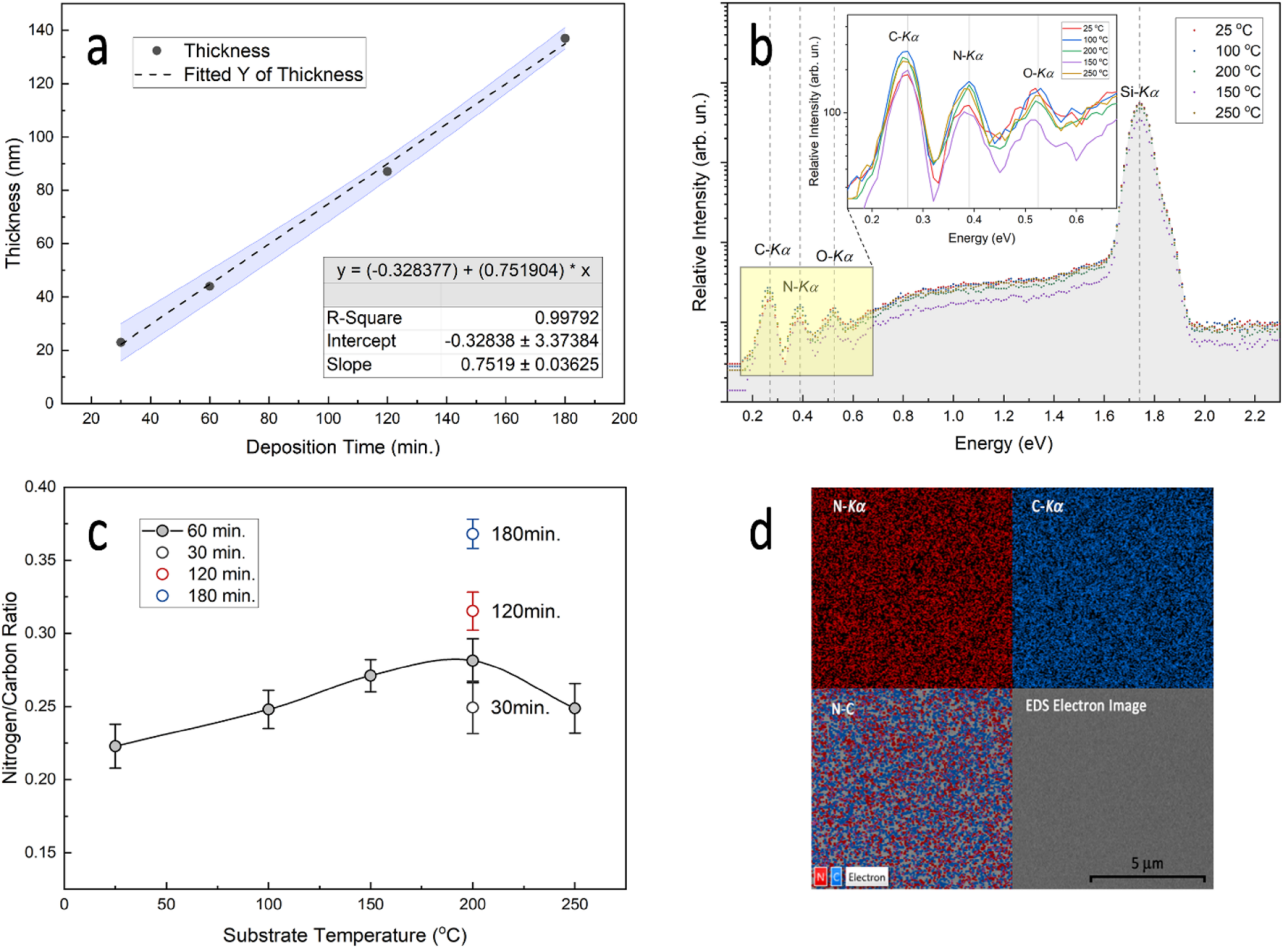
(**a**) Variation of thickness with deposition time for CNx films deposited at 200 °C. The linear regression fit parameters are shown as an inset, and the error bars are shown as filled areas. (**b**) Energy dispersive X-ray spectra for the films at different deposition temperature. (**c**) Variation of N/C ratio with substrate temperature and deposition time. (**d**) Energy dispersive X-ray spectroscopy mapping for the CNx film deposited at 200 °C and 120 min.

As we have previously mentioned, the optimum optical performance of the ARCs for solar cells is highly dependent on the precise control of film thickness and refractive index, as well as the morphology of the coating layer. The surface morphology and fractured cross-sections (as shown in the inset of Fig. 2) of the carbon nitride thin films prepared at different deposition times (30, 60, 120, and 180 min.) on silicon substrates at 200 °C are shown in Fig. 2. From the FESEM images, it can be seen that the surface morphologies of the as-deposited films on the silicon substrate were composed of very homogeneous fine-sized grains uniformly distributed over the film surface. For films deposited at of 30 min., the films exhibit an aggregation of grains distributed over an island structure, which indicates that the microstructure is in an early stage of film growth^53,56^. This is thus the threshold time at which the sputtered carbon nitride films have homogeneous surface morphology. When the deposition time is increased further, the surface structure becomes smoother and more densely packed with grains, with a corresponding increase in the film thickness. An obvious variation in the thickness and surface morphology of the film with deposition time was observed. The thickness of the film increased quite linearly from 23 to 137 nm as the deposition time increased from 30 to 180 min. Figure 3a correlates the resulting thicknesses with the deposition time. A linear regression fit with a slope of around 0.75 ± 0.04 nm/min can be extracted, as can be seen in the figure.

To investigate the concentration of nitrogen and carbon, EDS analysis was used to perform a quantitative analysis of the deposited carbon nitride films on a silicon substrate at different substrate temperatures. The results are illustrated in Fig. 3b, where the inset represents a zoomed section of the EDS spectra for the films in order to illustrate the X-ray lines of C:Kα and N:Kα, which are located at energies of 0.277 keV and 0.392 keV, respectively.

The figure shows that the film consists of mostly carbon and nitrogen, with a small amount of oxygen contamination. Based on the experimental EDS results, a plot of the nitrogen atomic concentration to carbon (N/C ratio) against the substrate temperature and deposition time is shown in Fig. 3c. Non-linear correlations between the N/C ratio and substrate temperature have been observed. The N/C ratio ranges from 0.22 for the film deposited at room temperature to a maximum of 0.28 for the film deposited at 200 °C, which implies that the deposited film is not a stoichiometric crystalline carbon nitride. Additionally, when the film was deposited at a higher temperature of 250 °C, a minor drop in the N/C ratio was recorded, suggesting that an increase in the substrate temperature may promote the desorption of the nitrogen atoms from the film surface. Interestingly, Fig. 3c illustrates that when deposition time was increased at a fixed deposition temperature, the N/C ratio increased significantly. The N/C ratio increased from 0.25 for the film deposited at 30 min. to about 0.37 for the film deposited at 180 min. Increases in deposition time result in increased plasma emission of nitrogen at the cost of C ions. The deposition process enters a nitriding state when nitrogen ion flux dominates with increasing sputtering time, reducing reactive C ion emission even more. The nitriding mode is generally induced by the surface poisoning of the target material, which results in a coating of carbon nitride on the graphite target, and this condition also results in a low emission of C ions^57,58^. The EDS chemical elemental mapping of CNx film is also shown in Fig. 3d, which is indicative of a highly uniform distribution of nitrogen and carbon elements over the surface of the film and shows no obvious surface segregation or agglomeration during deposition.

**Figure 4:**
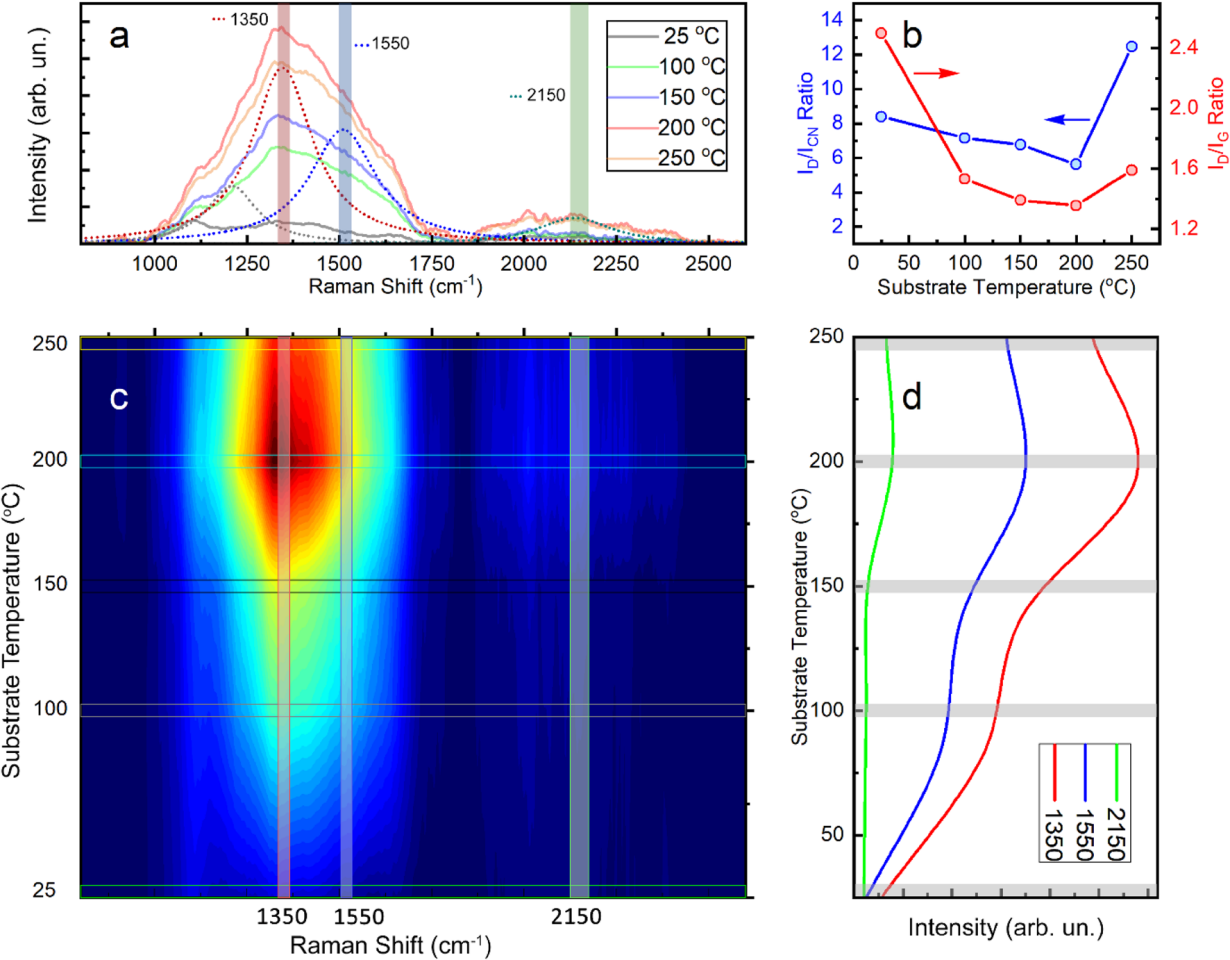
Contour profile Plot for the Raman spectra of the CNx films deposited on glass at different substrate temperatures. (**a**) Overlapping Raman spectra at different deposition temperatures, with deconvolution of the characteristic vibrational peaks (dot lines). (**b**) Variation of I_D_/I_G_ and I_D_/I_CN_ ratios as function of substrate temperature. (**c**) Generated contour map for Raman spectra at different temperatures. (**d**) Intensity of the three characteristic vibrational bands (***D***@1350 cm^−1^, ***G***@1550 cm^−1^, and ***CN***@2150 cm^−1^) in CNx films with substrate temperature.

**Figure 5:**
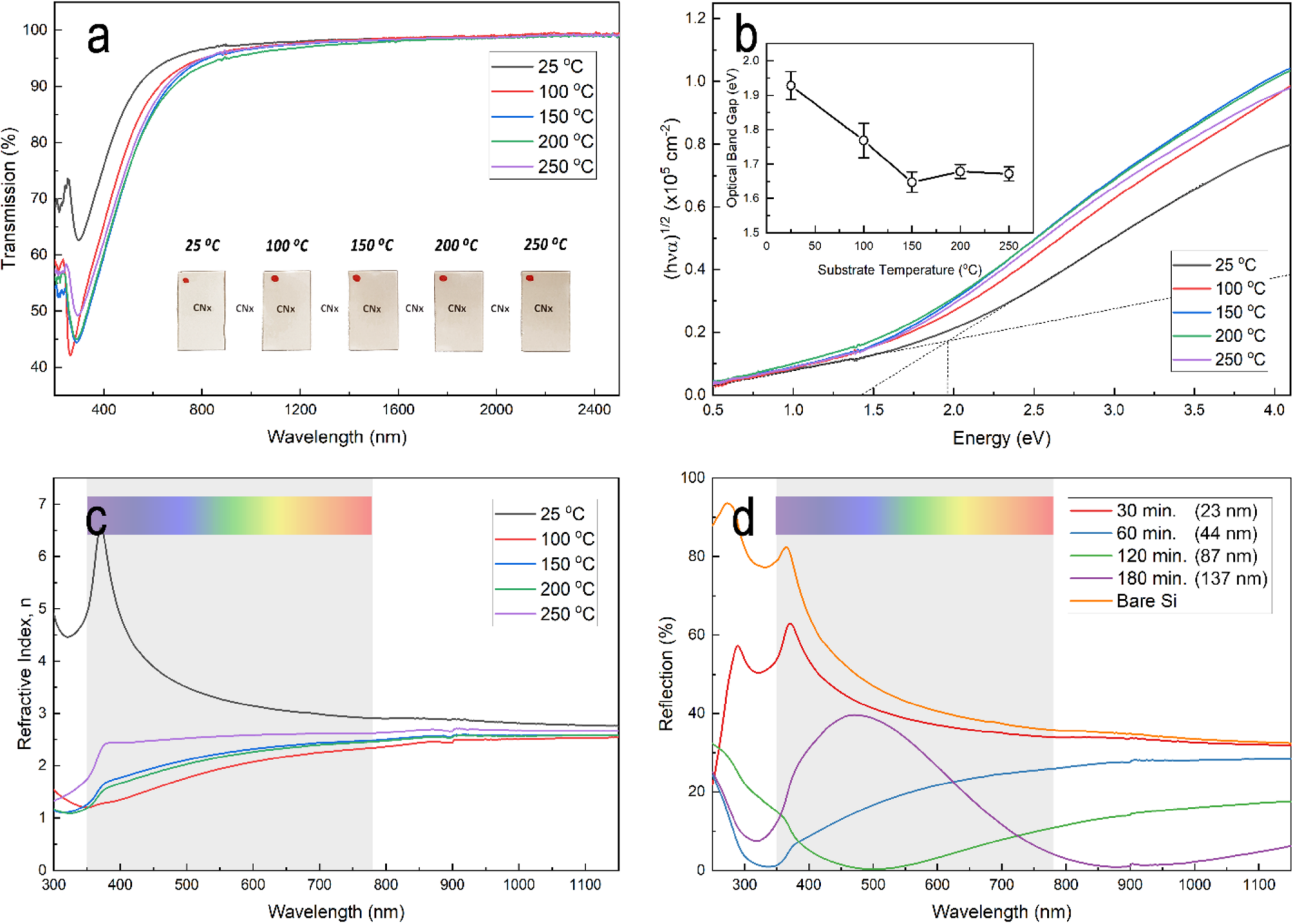
Optical properties of the CNx films deposited at various substrate temperatures and deposition time. (**a**) Optical transmission spectra for the films deposited on glass substrates at different substrate temperatures, with an inset showing the visible light transparency of the films. (**b**) Tauc plots of CNx films deposited at various substrate temperatures. The inset represents the variation of the optical band gap with substrate temperature. (**c**) Refractive index as a function of wavelength for the CNx films deposited at various substrate temperatures. (**d**) Reflection spectra for films deposited at 200 °C and different deposition times (layer thickness), with a reference reflection of a bare silicon wafer.

The contour profile for the Raman spectra of the carbon nitride films deposited at different substrate temperatures is plotted in Fig. 4. The overlapped Raman spectra of the CNx films in Fig. 4a are fairly consistent with the typical spectrum of the amorphous carbon film. The Raman spectra of the CNx films are typically composed of (***D***) band at 1350 ± 15 cm^−1^ and (***G***) band at 1550 ± 15 cm^−1^ and are also common bands for disordered carbon materials such as DLC^59–61^. In addition to these two bands, other bands were also observed at around 2150 ± 50 cm^−1^, which is related to the nitrogen-carbon triple bond (***CN*** band). The most common atomic bonding configurations for carbon and nitrogen in the CNx structure are linear, trigonal, and tetrahedral, which correspond to sp^1^, sp^2^, and sp^3^ hybridizations, respectively^62^. The ***D*** band originates from the breathing vibrational modes of sp^2^ hybridization of carbon atoms in sixfold ring clusters, and becomes active in disordered graphite-like structures. The ***G*** band arises from the in-plane stretching vibrational modes of sp^2^ hybridization in both six-fold rings and chains of carbon atoms. Deconvoluting the Raman spectra into three Gaussian peaks, the ***D*** band, the ***G*** band, and the ***CN*** band, is shown in Fig. 5a, while Fig. 5b shows the intensity ratio of (I_D_/I_G_) and (I_D_/I_NC_) as a function of substrate temperature, and Fig. 5d illustrates the variation of intensities of the three peaks with the deposition temperature. As carbon nitride films were deposited at 200 °C, the smallest peak ratio ID/IG ratio was observed. With a further increase in substrate temperature, the I_D_/I_G_ ratio becomes higher. The value of the I_D_/I_G_ ratio is related to the amount of the structural disorder produced by nitrogen incorporation in CNx films. The variation of I_D_/I_NC_ ratio with increasing substrate temperature showed a similar trend to that of I_D_/I_G_ ratio. Generally, a high I_D_/I_G_ ratio is an indication for a high content of sp^2^ carbon clusters^60,63,64^. As a consequence, the amount of sp^2^ carbon clusters reduced as the substrate temperature increased up to 200 °C. However, further increasing the substrate temperature resulted in the sp^3^ hybridization transforming into the graphitic structure. The high temperature can enhance the formation of sp^3^ hybridization at the surface layer of the films. When temperature is increased, the sp^3^ transforms into sp^2^ hybridization, potentially due to thermally activated diffusion^65,66^. Another possible explanation for the lower I_D_/I_G_ ratio at higher temperatures is the reduced nitrogen concentration in the CNx films, as previously shown by EDS analysis.

The optical properties of the films were determined from optical transmission and reflection measurements. Figure 5a shows the optical transmission spectra of the CNx film deposited at different substrate temperatures. The spectra clearly show that the film has a high optical transmission in the range of 200–2500 nm for all the CNx films. However, films deposited at a lower temperature are shown to be much more transparent than films deposited at a higher temperature (see the inset for the sample photos). The transmission of the film prepared at room temperature was 91% at 550 nm. Whereas the optical transmission of the film prepared at 200 °C decreased to 81% at 550 nm. The films have a transmission of between 52–96% in the visible region (350–780 nm) and higher than 89% in the near-infra-red (NIR) region, which makes them a good potential candidate for NIR device applications. The results suggest that this behavior is related to a band structure alteration in the deposited CNx films. The high transmission implies that the carbon and nitrogen atoms of the sp^3^ fraction are dominant in the films deposited at a low substrate temperature. A lower transmission was observed for the films prepared at higher temperatures, with a significant role for graphitic sp^2^ clustering, as discussed in the previous section.

The transmission spectra of the films deposited on glass substrates are used to estimate the optical energy gaps (E_g_) of the CNx films. According to the well-known Tauc relation^67^, the optical gap, E_g_, is determined by extrapolating the linear relation of (*αhν*)^1/2^ versus photon energy *hν* (Fig. 5b), where *α* is the absorption coefficient, *ν* is the frequency of incident photons, and *h* is the Planck's constant. The optical gap of the films decreases with increasing substrate temperature, as illustrated in the figure inset. The calculated optical gaps for the films prepared at 25, 100, 150, 200, and 250 °C were 1.93 eV, 1.77 eV, 1.65 eV, 1.68 eV, and 1.67 eV, respectively. Results confirm that the increase in nitrogen content in the film structure causes an increase in the optical energy gap. This result, however, is consistent with previous work on CNx films and DLC films, in which the band gap was found to be strongly dependent on the I_D_/I_G_ ratio as well as sp^2^ clustering^68–70^.

Figure 5c shows the variation of the refractive index of CNx films deposited on glass at different substrate temperatures over the spectral range of 300–1200 nm. As shown in the figure, the refractive index decreases with increasing substrate temperature. As the substrate temperature was increased from room temperature to 100 °C, the refractive index at 550 nm dropped from 3.28 to 1.94. When the temperature of the substrate is increased further, the refractive index slightly increases to 2.56. The effect of photon scattering of nitrogen and carbon atoms is commonly associated with determining the refractive index of the CNx films. Thus, the structural development shown by the Raman and EDS spectra discussed above may account for the variations in the refractive index as the substrate temperature increases. Generally, the more nitrogen incorporation and more sp^2^ clusters in the films, the lower the refractive index is^71^. When the substrate temperature is increased from room temperature to 100 °C, the increasing nitrogen content in the CNx films results in a decrease in refractive index, and the decreasing sp^3^ clusters result in an even greater decrease in refractive index as the substrate temperature is increased further. The behavior of the refractive index as substrate temperature increased was comparable to that described in previous works^72,73^.

In order to achieve the minimum reflectance of the surface surrounded by a homogenous medium with a different refractive index, precise control of the thickness and refractive index of the coating material is essential. Generally, the refractive index and coating thickness must meet the following conditions to minimize the reflectance of the substrate to zero at a specific wavelength (λ)^4,44,48^:

1 $${n}_{c}=\sqrt{{n}_{s}.{n}_{a}}\,\,\,{\text{and}}\,\,\,{t}_{c}=\lambda /(4\times {n}_{c})$$

where *n*_*c*_, n_s_, and *n*_*a*_ are the refractive index of the ARC, substrate, and air, respectively, and t_c_ is the thickness of the coating film. The conditions are valid only for a transparent and homogeneous medium, as the absorbing materials may add more complications to the equation due to the loss. Since the refractive index of crystalline silicon used for solar energy applications is around 4, any ideal ARC material must have a refractive index value of around 2 as per Eq. (1). The current commercial example of such an efficient ARC material is silicon nitride, with a refractive index of 2.02 in the middle of the visible spectrum at 550 nm. With a 68 nm thickness, Si_3_N_4_ can reduce the average reflectance in the visible spectrum to about 5.3%^74^.

The reflectance spectra of polished bare silicon and CNx films on silicon substrates with different deposition times is shown in Fig. 5d. As with the ARC-coated silicon substrates, the surface reflectance was significantly reduced, indicating that the CNx coatings have superior anti-reflection properties. The reflectance curve for the CNx film deposited at 120 min has a typical wide U-shape curve with a low reflectance of less than 0.3% at 550 nm, compared to roughly 47% for the polished bare silicon at the same wavelength. The minimum reflectance was observed at a wavelength of 500 nm, which is slightly shifted from the central wavelength of visible light. However, this may be adjusted experimentally by optimizing the refractive index and thickness of the deposited CNx films. The average reflectivity of the ARC film for the wavelength range of 300–1100 nm was approximately 9.8%. For the longer deposition time of the CNx films, the reflectance curves also show broad antireflective performance in the NIR region, where the CNx films have a very low reflectivity of 1% at 900 nm.

The water contact angle (WCA) formed by a small drop of water on the surface of a substance is a measure of the surface energy of that substance. When the material has low surface energy, the WCA will be wide. When the WCA exceeds 90°, the surface is considered hydrophobic. A superhydrophobic coating with a large WCA (greater than 150°) is highly desirable for antireflective coatings applied to solar cells, as such coatings have the ability to roll off the surface and hence clean any contaminants along the way, resulting in a self-cleaning surface.

**Figure 6:**
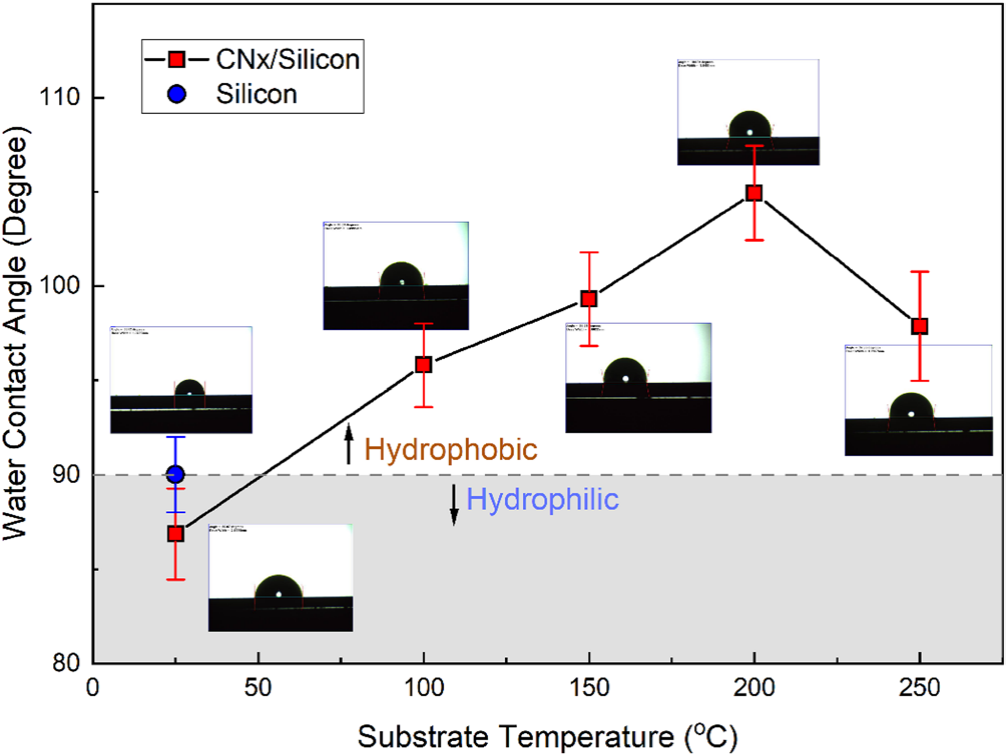
The static water contact angle WCA of bare silicon and CNx films deposited at different substrate temperatures (the insets represent the photos of water droplets on the film surface).

Figure 6 shows the WCA of CNx films deposited at various substrate temperatures, in addition to the bare silicon sample which has been used as a reference. It is observed that all the CNx films show excellent hydrophilicity, except for the film deposited at room temperature. With increasing substrate temperature from RT to 200 °C, the WCA increased from 87° to 105°. With a further increase in substrate temperature, the WCA decreased slightly to 89°. The results show that the largest WCA recorded for the CNx film was deposited at 200 °C. Recent research reported that the CNx film surface has hydrophobicity or even superhydrophobic characteristics. The wetting of the CNx thin films is sensitive to the sp^2^ and sp^3^ fractions in the structure as have been reported previously^75,76^. Reports indicate that films with high sp^2^ clusters exhibit higher WCA than films with higher sp^3^ clusters. Thus, the improvement in WCA of CNx thin films as the substrate temperature increases might be related to an increase in sp^2^ clustering, as shown by Raman analysis.

**Figure 7:**
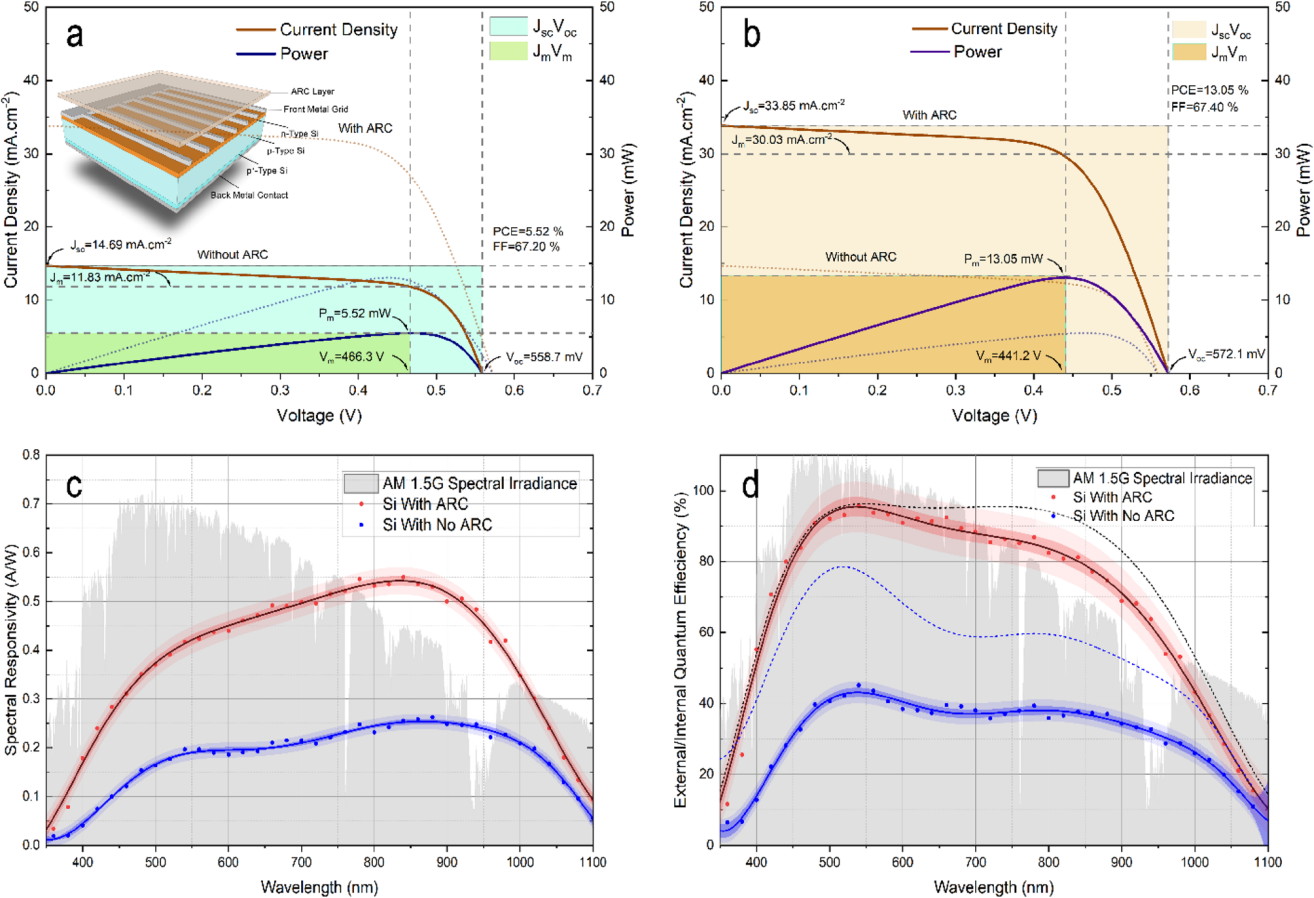
Optoelectrical performance of the crystalline silicon solar cell with and without CNx anti-reflective coating. (**a**) The current density–voltage curve of an uncoated c-Si solar cell. The inset is a schematic of the structure of the cell. (**b**) Current density–voltage curve of a CNx-coated c-Si solar cell. The dot lines for the uncoated c-Si solar cell. (**c**) Spectral responsivity for the c-Si solar cell with and without ARC of CNx. (**d**) The external/internal (dash lines) quantum efficiency of a c-Si solar cell with and without CNx anti-reflective coating.

The CNx films showed optical properties that make it a potential candidate for their use as an efficient ARC in a c-Si solar cell application. The film was deposited over commercial c-Si solar cells at a deposition time and temperature of 120 min and 200 °C, respectively. Figure 7a,b display the current density versus voltage characteristic of a commercial c-Si with and without CNx coating, respectively. The measurement was carried out under AM1.5G illumination with an intensity of 100 mWcm^−2^. The basic performance parameters of the solar cell, including short-circuit current density (Jsc), open circuit voltage (Voc), fill factor (FF), and photoelectric conversion efficiency (PCE), are also shown in the figures. A significant enhancement of the Jsc and PCE was observed after coating the c-Si solar cell with the CNx layer. The Jsc was increased from 14.69 to 33.85 mAcm^−2^ and the PCE was increased from 5.52 to 13.05%. The improvement in PCE is mostly related to the improvement in the Jsc. In principle, the effect of the Jsc is reflects the actual efficiency of the ARC, since it is directly related to the reduction of the surface reflectance and hence increases the photocurrent generated by the photons absorbed in the solar cell. We notice that after applying a CNx coating to the c-Si solar cell, Voc increased slightly from 559 to 572 mV, which suggests the possibility of other effects that might contribute to the increase in the Voc, such as the front-surface passivation effect^77^. Additionally, the FF of the solar cell coated with a CNx layer was almost equal to that of the uncoated cell, since the FF is related to the series and parallel resistance of the cell, which is unaffected by the ARC layer applied.

Based on the spectral responsivity (Fig. 7c) and external/internal quantum efficiency results (Fig. 7d), it is clear that the CNx coating on the c-Si solar cell results in a significant improvement in the external quantum efficiency over a wide range of wavelengths from 300 to 1100 nm. The maximum spectral responsivity rose from about 0.27 to about 0.57 AW^−1^, and the external quantum efficiency improved from 46 to 98%. The spectral responsivity plot revealed that the peak of response was found to be at 850 nm due to the absorption edge of the silicon substrate. Decreasing the responsivity after 850 nm can be attributed to the cut-off wavelength of silicon^78^. The maximum observed external quantum efficiency was centered at a wavelength of 500 nm, which was expected from the optical reflectance results for the CNx coated silicon wafer.

Table 1 summarizes the optical characteristics and performance parameters of the reported antireflective coating on crystalline silicon solar cells and the results of the present work. It was observed that the improvement of silicon solar cell parameters using conventional SiNx and DLC coating was almost comparable. However, the results achieved with CNx coatings were much superior than those obtained with both coatings, e.g., reflectance was reduced by about 78% in the wavelength range of 300–1100 nm, resulting in the greatest enhancement in the c-Si solar cell efficiency. Besides the outstanding anti-reflective properties, the CNx coating exhibits good hydrophobic properties, making it an ideal candidate for anti-reflection coatings on high-efficiency c-Si solar cells.

**Table 1:** Summary of the average reflectance, EQE, and PCE of silicon solar cells with different single layer ARC materials compared with the CNx ARC of the present work (*).

ARC material	Thickness (nm)	Average reflectance (%)	Improvement (%)	Average EQE (%)	Improvement (%)	PCE (%)	Improvement (%)	Ref.
SiNx	80	13.56	63.6	74.69	31.5	16.14	32.6	^29^
SiNx	80	12.64	26.7	60.22	22.3	17.60	38.6	^35^
SiNx	80	12.54	65.0	–	–	14.25	–	^36^
DLC	80	8.51	50.6	63.29	28.5	18.40	44.9	^35^
DLN	85	14.90	58.4	–	–	13.67	–	^36^
DLC	86	16.04	63.1	–	–	9.00	38.0	^37^
CNx	87	9.71	78.0	67.91	108.3	13.05	136.4	*

The average was calculated in the wavelength range of 300–1100 nm.

### Thin films deposition

CNx ARC films were prepared on glass, silicon, and commercial c-Si solar cell substrates using Mantis NanoSys500 multi PVD system with a 2-inch diameter and 3 mm thick graphite target from Kurt J. Lesker (purity of 99.999%). The silicon substrates used in this study were chemically treated with diluted HF solution to remove the native oxide layer, and all substrates were ultrasonically cleaned in an organic solvent bath, thoroughly rinsed with ethanol, acetone, and deionized water, and dried with an N_2_ flow. The c-Si solar cell was made of 500 μm thick untextured boron doped silicon wafer with a resistivity of 5 Ω cm and 0.3 μm depth front junction diffusion layer with a doping concentration of about 1 × 10^−18^ cm^−3^. The system chamber was first pumped down to a base pressure of about 10^−7^ mbar prior to each deposition run. The nitrogen gas flow rate was kept constant at 50 sccm for all the samples through a mass flow controller, so that the total vacuum level in the main chamber was maintained at about 2.5 × 10^−2^ mbar. The deposition rate of the films was determined by a Prevac TMC13 quartz crystal thickness monitor and the film thickness were later verified by SEM images of the film cross section. The ARC films were deposited at different temperatures: 25, 100, 150, 200, and 250 °C using different deposition times: 30, 60, 120 and 180 min. and at a constant substrate/target distance of 15 cm. To achieve high-uniformity nitride films, the substrate was constantly rotated at 5 rpm during the deposition process.

### Samples characterizations

The surface morphology of ARC films was investigated by a field emission scanning electron microscope (FESEM, Mira III, TESCAN, Czech Republic) equipped with an energy dispersive X-ray spectroscopy detector (EDS, X-MAX, Oxford Instruments, UK) for analysis of the elemental composition of the films. The topography of the film surfaces was measured by an atomic force microscope (AFM, Ntegra, NT-MDT, Russia). The transmission and reflectance of ARC films on glass and silicon substrates were measured by a UV/Vis–NIR double beam spectrophotometer (V-670, JASCO, Japan). The instrument was equipped with a reflectance measurement accessory. Raman analysis was performed using the Raman microscope (Sentera, Bruker Optics, Germany) with an excitation laser wavelength of 523 nm. The water contact angles of the bare p-type silicon substrate and the ARC sample were measured with a sessile drop analyzer to evaluate their wettability performance.

## Data Availability

The datasets generated during and/or analysed during the current study are available from the corresponding author (A.J.A.) on reasonable request.
